# Phytocannabinoid-drug interactions: pharmacokinetic and pharmacodynamic mechanisms, clinical relevance and toxicological considerations

**DOI:** 10.3389/ftox.2026.1952599

**Published:** 2026-09-28

**Authors:** Diogo Alexandre Gomes, Wellyngton Vieira Eufrázio, Marcella Silva Ribeiro, Linério Ribeiro de Novais Júnior, Flávia Rigo, Patrícia de Aguiar Amaral, Antonella Campanale, Antônio Inserra, Rafael Mariano de Bitencourt

**Affiliations:** 1 Behavioral Neuroscience Laboratory, Postgraduate Program in Health Sciences, University of Southern Santa Catarina (UNISUL), Tubarão, Brazil; 2 Medicinal Plant Laboratory (LaPlaM), Postgraduate Program in Health Sciences, University of the Extreme South of Santa Catarina (UNESC), Criciúma, Brazil; 3 Canadian Consortium for the Study of Natural Psychedelics and Consciousness (CCNPC), Montreal, QC, Canada; 4 Centre for Behavioural Sciences and Mental Health, Istituto Superiore di Sanità (ISS, Italian National Institute of Health), Rome, Italy; 5 Department of Neuroscience, Imaging and Clinical Sciences, G. D’Annunzio University of Chieti Pescara, Chieti, Italy

**Keywords:** cannabidiol, cytochrome P450, drug-drug interactions, pharmacokinetics, phytocannabinoids, toxicology, Δ9-tetrahydrocannabinol

## Abstract

The therapeutic use of phytocannabinoids has expanded considerably over the past decade because of their potential application across a wide range of clinical conditions. However, their concomitant use with conventional medications raises important concerns regarding drug interactions and patient safety. This narrative review provides a critical overview of current evidence on the pharmacokinetic (PK), pharmacodynamic, and toxicological interactions of major phytocannabinoids, with particular emphasis on cannabidiol (CBD) and Δ^9^-tetrahydrocannabinol (THC), while also discussing emerging evidence related to minor phytocannabinoids and the potential contribution of terpenes. Phytocannabinoids modulate the activity of drug-metabolizing enzymes, particularly cytochrome P450 isoenzymes, and may affect selected drug transporters, thereby altering the systemic exposure and clinical effects of co-administered medications. Conversely, conventional medications that inhibit or induce cannabinoid-metabolizing enzymes may modify phytocannabinoid exposure, efficacy, and tolerability. Documented or potentially clinically relevant interactions have been reported for antiseizure medications, anticoagulants, antidepressants, opioids, alcohol, and other central nervous system depressants. However, the strength of the supporting evidence varies considerably, ranging from *in vitro* predictions and case reports to controlled PK and clinical studies. The magnitude and clinical relevance of these interactions are influenced by multiple factors, including cannabinoids formulation, dose, route of administration, treatment duration, pharmacogenetic variability, patient-specific characteristics, and polypharmacy. Particular attention should be given to vulnerable populations, such as pregnant women, children, older adults, and patients with hepatic impairment, in whom altered PK and increased susceptibility to adverse effects may further complicate treatment. Although substantial progress has been made in understanding cannabinoid pharmacology, important knowledge gaps remain regarding the clinical significance of many predicted interactions, the interaction profiles of minor phytocannabinoids, full-spectrum preparations, and terpenes and the long-term safety of cannabinoid-based therapies. Careful differentiation between mechanistically plausible and clinically demonstrated interactions, together with consideration of cannabinoid composition, dose, route of administration, patient vulnerability, and concomitant medications, can support safer and more individualized clinical management while guiding future research priorities.

## 1 Introduction

*Cannabis sativa* has been used for medicinal purposes for thousands of years and has attracted increasing scientific and clinical interest in recent decades due to the growing body of evidence supporting the therapeutic potential of its bioactive constituents. Phytocannabinoids have shown promising effects in the management of several clinical conditions, including chronic pain, epilepsy, sleep disorders, anxiety, and other neurological and neuropsychiatric disorders (Bitencourt et al., 2021; Greis et al., 2022; Hill et al., 2023; Hidding et al., 2024; Caicedo et al., 2025). As their therapeutic use expands, phytocannabinoids are increasingly administered to patients receiving concomitant pharmacological treatments, making the evaluation of potential drug–drug interactions an important clinical and safety consideration.

The expanding clinical use of cannabinoid-based products has been accompanied by increasing concerns regarding their safety, particularly when administered concomitantly with conventional medications. Phytocannabinoids may alter the pharmacokinetic (PK), pharmacodynamic (PD) of numerous drugs through modulation of drug-metabolizing enzymes, transport proteins, and intracellular signaling pathways (Stout and Cimino, 2014; Zendulka et al., 2016; Balachandran et al., 2021; Nachnani et al., 2024), thereby affecting systemic drug exposure, therapeutic efficacy, and the risk of adverse drug reactions (Brown and Winterstein, 2019; Balachandran et al., 2021). Conversely, conventional medications that inhibit or induce enzymes involved in cannabinoid metabolism may alter phytocannabinoid exposure, efficacy, and tolerability (Grotenhermen, 2003; Stout and Cimino, 2014). Although considerable progress has been made in understanding these mechanisms, important gaps remain regarding the clinical relevance of many reported and predicted interactions and the influence of factors such as cannabinoid formulation, phytochemical composition, dose, route of administration, treatment duration, pharmacogenetic and other patient-specific variability, and polypharmacy (Abuhasira et al., 2018; Gottschling et al., 2020; Nachnani et al., 2024).

In this context, this narrative review aims to provide a comprehensive and critical overview of the PK, PD, and toxicological interactions between phytocannabinoids and conventional medications. Particular emphasis is placed on Δ9-tetrahydrocannabinol (THC) and cannabidiol (CBD), while emerging evidence concerning minor phytocannabinoids, terpenes, and different *Cannabis*-derived formulations is also considered. Unlike previous reviews focused primarily on PK interactions between cannabis products and medications, this review integrates PK, PD, and toxicological evidence; evaluates major and minor phytocannabinoids and formulation-related variability; distinguishes predicted interactions from those demonstrated clinically; and considers implications for vulnerable populations, polypharmacy, and individualized clinical management.

## 2 Literature search and evidence synthesis

This narrative review was informed by structured literature searches conducted in PubMed/MEDLINE, Embase, and Scopus from database inception through 22 July 2026. Search strategies combined controlled vocabulary (where applicable) and free-text terms related to phytocannabinoids, cannabinoids, *Cannabis*-derived products, cannabidiol (CBD), Δ^9^-tetrahydrocannabinol (THC), drug-drug interactions, PK, PD, drug-metabolizing enzymes, transport proteins, toxicity, and safety. Additional targeted searches were conducted to identify evidence regarding clinically relevant medications and therapeutic classes, including antiepileptic drugs, anticoagulants, immunosuppressants, opioids, benzodiazepines, antidepressants, antipsychotics, and cardiovascular medications. Reference lists of relevant original studies, systematic reviews, and narrative reviews were also screened to identify additional pertinent publications.

The core search structure combined cannabinoid-related terms (phytocannabinoid*, cannabinoid*, *Cannabis*, cannabidiol OR CBD, and tetrahydrocannabinol OR THC) with interaction- and safety-related terms (drug interaction*, pharmacokinetic*, pharmacodynamic*, CYP, cytochrome P450, UGT, transporter*, toxicity, safety, and adverse effect*). Additional medication- and therapeutic-class terms were then applied in targeted searches. Search syntax was adapted to the controlled vocabulary and indexing conventions of each database.

The search was intended to identify representative mechanistic and clinical evidence rather than provide exhaustive systematic coverage. Priority was given to human clinical studies, systematic reviews, meta-analyses, and mechanistic investigations, while selected preclinical studies were included when they provided important mechanistic insights or addressed evidence gaps not yet investigated clinically.

Studies were considered eligible when they directly addressing phytocannabinoid–drug interactions or interaction-relevant mechanisms, including controlled human clinical and PK studies, observational studies, case reports or case series, PBPK or other computational studies, *in vitro* studies using human or experimental systems, and selected animal studies when they provided mechanistic information not yet available in humans. Studies were not considered further when they did not address phytocannabinoid or *Cannabis* exposure, a co-administered medication or clinically relevant interaction mechanism, or PK, PD, or toxicological outcomes relevant to the topic of the review. Because this was a narrative rather than systematic review, no formal risk-of-bias score or meta-analytic evidence grade was assigned.

Evidence was categorized according to its source as (i) controlled clinical studies, (ii) dedicated human PK interaction studies, (iii) prospective observational studies, (iv) case reports or case series, and (v) preclinical, PBPK/*in silico*, or *in vitro* studies. Clinical interpretation considered the directness of the evidence to human co-administration, consistency and replication of findings, magnitude of the observed effect, relevance of cannabinoid dose, formulation and route of administration, and whether the reported findings were linked to clinically meaningful outcomes or primarily to mechanistic plausibility. These categories were intended to facilitate interpretation of the available literature rather than establish a rigid hierarchy of methodological quality, as different study types can provide complementary and clinically relevant information.

## 3 Endocannabinoid system and phytocannabinoids

### 3.1 Endocannabinoid system

The endocannabinoid system (ECS) is a regulatory network comprising cannabinoid receptors, endogenous ligands—primarily anandamide (AEA) and 2-arachidonoylglycerol (2-AG)—and the enzymes responsible for their synthesis and degradation. It helps maintain physiological balance, or homeostasis, by regulating mood, stress responses, pain, appetite, sleep, memory, immunity, metabolism, and gastrointestinal function (Moreira and Lutz, 2008; Cristino et al., 2019). It also contributes to communication between the nervous, endocrine, and immune systems (De Laurentiis et al., 2022). The two principal cannabinoid receptors, the CB1 and CB2 receptors, exhibit distinct tissue distributions, with CB1 being predominantly expressed in the central nervous system (CNS) and CB2 primarily associated with immune cells and peripheral tissues (Lu and Mackie, 2016; Navarro et al., 2018).

The classical definition of the ECS has subsequently expanded into the concept of the endocannabinoidome (eCBome), which encompasses a broader network of endocannabinoid-related lipid mediators, their anabolic and catabolic enzymes, and molecular targets beyond CB1 and CB2. These include transient receptor potential channels, peroxisome proliferator-activated receptors, and several orphan G protein-coupled receptors (Di Marzo, 2020). The eCBome therefore provides a more comprehensive framework for understanding the physiological functions of endocannabinoid-related signalling and the complex pharmacological actions of phytocannabinoids.

The characterization of the eCBome has substantially expanded the therapeutic interest in phytocannabinoids, particularly THC and CBD. THC exerts most of its pharmacological effects through partial agonism of CB1 and CB2 receptors, whereas CBD displays low affinity for these receptors and acts through multiple receptor-dependent and receptor-independent mechanisms, including modulation of transient receptor potential vanilloid 1 (TRPV1), serotonin 5-HT1A receptors, G protein-coupled receptor 55 (GPR55), peroxisome proliferator-activated receptor gamma (PPARγ), and fatty acid amide hydrolase (FAAH). These diverse molecular targets contribute to the broad therapeutic profile of CBD and help explain its potential application in the management of chronic pain, sleep disorders, anxiety, epilepsy, inflammatory diseases, and other neurological and neuropsychiatric conditions (Lu and Mackie, 2016; Elanne et al., 2020).

Beyond providing the biological basis for the therapeutic effects of phytocannabinoids, advances in the understanding of the eCBome have also highlighted the complexity of cannabinoid pharmacology and the need to better characterize their interactions with conventional medications. In addition to receptor-mediated effects, phytocannabinoids can influence drug disposition by modulating metabolizing enzymes, membrane transporters, and intracellular signaling pathways, thereby affecting the PK and PD of co-administered drugs. Consequently, understanding the relationship between phytocannabinoids and the eCBome is essential not only for the development of novel therapeutic strategies but also for optimizing the safety, efficacy, and individualized use of cannabinoid-based therapies in clinical practice (Bruni et al., 2018; Navarro et al., 2018; Zou and Kumar, 2018).

### 3.2 Major phytocannabinoids

Phytocannabinoids are naturally occurring compounds found in *Cannabis sativa* that exert diverse pharmacological effects through interactions with the eCBome. More than 200 phytocannabinoids have been identified to date, although only a limited number have been extensively investigated regarding their pharmacological properties, therapeutic potential, and interaction profiles (Hanuš et al., 2016; ElSohly et al., 2017; Chalannavar et al., 2026).

THC produces analgesic, antiemetic, orexigenic, and antispastic effects, supporting its therapeutic use in several clinical conditions (Bonfá et al., 2008; Machado Rocha et al., 2008). However, THC-mediated activation of CB1 receptors within the CNS also explains many of its adverse effects and PD interactions, including additive sedation and cognitive impairment when co-administered with benzodiazepines, opioids, alcohol, and other CNS depressants (Balachandran et al., 2021; Breijyeh et al., 2021).

CBD, one of the most abundant phytocannabinoids, lacks psychotomimetic properties and has attracted considerable interest because of its broad therapeutic potential. Clinical evidence supports its use in treatment-resistant epilepsy, while growing evidence suggests potential benefits for anxiety disorders, chronic pain, neuroinflammatory conditions, and other neurological diseases (Schier et al., 2012; Bitencourt and Takahashi, 2018; Freeman et al., 2019; Silvestro et al., 2019; Bitencourt et al., 2021). Unlike THC, CBD exhibits low affinity for CB1 and CB2 receptors and exerts its biological effects through multiple molecular targets. Of particular relevance to the present review, CBD can inhibit several hepatic drug-metabolizing enzymes, especially cytochrome P450 and uridine 5′-diphospho-glucuronosyltransferase (UGT) isoforms, thereby increasing the potential for clinically relevant drug-drug interactions (Bansal et al., 2020; Smith and Gruber, 2023).

Among the minor phytocannabinoids, cannabigerol (CBG) has demonstrated neuroprotective, anti-inflammatory, antioxidant, and potential ocular hypotensive effects, making it a promising candidate for neurological disorders and glaucoma (Granja et al., 2012; Morrone et al., 2015; Calapai et al., 2022). Nevertheless, current knowledge regarding its PK profile and interaction with conventional medications remains limited (Navarro et al., 2018; Nachnani et al., 2021).

Cannabinol (CBN), a degradation product of THC, has been suggested to possess mild sedative properties and has therefore attracted interest as a potential therapeutic agent for sleep disorders. However, clinical evidence supporting this indication remains scarce. Consequently, concomitant administration with other CNS depressants should be approached cautiously because of the lack of studies and potential for additive sedative effects (Babson et al., 2017).

Another phytocannabinoid receiving increasing attention is tetrahydrocannabivarin (THCV), which has demonstrated promising effects on energy metabolism, glucose homeostasis, and body weight regulation in preclinical studies (Abioye et al., 2020). However, its PK characteristics and potential drug interactions remain poorly characterized, requiring caution, while representing an important area for future investigation.

Although THC and CBD remain the most extensively studied phytocannabinoids, increasing evidence suggests that several minor cannabinoids and other bioactive constituents of *Cannabis* sativa, particularly terpenes and flavonoids, may also contribute to the overall pharmacological profile of *Cannabis* preparations. However, compared with CBD and THC, the available evidence regarding the pharmacology, PK, PD, and clinical interaction profiles of minor phytocannabinoids remains substantially limited, and many proposed interactions are based on mechanistic or preclinical evidence rather than clinical studies. Through their potential interactions with cannabinoids, these additional constituents may modulate pharmacological activity and influence therapeutic responses and safety profiles, a hypothesis commonly referred to as the entourage effect (Russo, 2011). Despite promising preclinical findings, robust clinical evidence supporting the therapeutic relevance of this phenomenon remains limited. Likewise, evidence regarding drug interactions involving minor phytocannabinoids remains predominantly preclinical or *in vitro*. Further clinical studies are therefore needed to establish both the magnitude and clinical significance of these interactions.

### 3.3 Terpenes and the entourage effect

Terpenes constitute one of the largest classes of secondary metabolites found in *Cannabis sativa*, contributing not only to the plant’s characteristic aroma but also to its pharmacological profile. Approximately 200 terpenes have been identified in *Cannabis sativa*, although only a limited number are consistently present in clinically relevant concentrations. Among the most abundant are β-caryophyllene, myrcene, limonene, α-pinene, and linalool, each exhibiting distinct biological activities, including anti-inflammatory, analgesic, anxiolytic, antioxidant, and neuroprotective properties (Russo, 2011; ElSohly et al., 2017).

The potential pharmacological interaction between phytocannabinoids and terpenes gave rise to the concept known as the entourage effect, originally proposed to describe synergistic interactions among the multiple bioactive constituents naturally present in *Cannabis sativa* (Russo, 2011). According to this hypothesis, the therapeutic effects of *Cannabis*-based preparations may result not only from the activity of individual cannabinoids but also from complex interactions involving terpenes, flavonoids, and other phytochemicals capable of modulating receptor signaling, neurotransmission, inflammation, and PK processes.

Experimental studies have suggested that individual terpenes may modulate specific pharmacological effects of phytocannabinoids. For example, myrcene has been associated with enhanced sedative and muscle-relaxant effects of THC, limonene with anxiolytic-like responses and mood improvement, linalool with calming properties that may complement the effects of CBD, α-pinene with a potential attenuation of THC-induced cognitive impairment, and β-caryophyllene with anti-inflammatory activity through CB2 receptor activation (Ashton, 2001; Russo, 2011; Nuutinen, 2018; Gonçalves et al., 2020; Vega-García et al., 2021). However, these observations are derived predominantly from preclinical investigations, and their clinical relevance remains to be established.

Although the entourage effect has become widely investigated, current evidence remains heterogeneous. Experimental studies support potential synergistic interactions between cannabinoids and terpenes; however, clinical studies demonstrating consistent therapeutic advantages of full-spectrum preparations over isolated cannabinoids remain limited (Ferber et al., 2020; Finlay et al., 2020). Therefore, despite increasing scientific interest, current evidence remains insufficient to establish the clinical relevance of the entourage effect, and further well-designed mechanistic and clinical studies are required.

From the perspective of phytocannabinoid–drug interactions, the entourage effect may have important implications. Variations in the phytochemical composition of *Cannabis* extracts can modify PD responses and potentially influence metabolic pathways involved in drug disposition. Consequently, differences between isolated cannabinoids and full-spectrum extracts should be considered when evaluating PK behavior, therapeutic responses, and the risk of clinically relevant drug interactions. However, direct clinical evidence demonstrating that terpene–cannabinoid interactions materially alter systemic drug exposure or clinically important interaction risk remains insufficient. These considerations reinforce the importance of investigating the PK characteristics of phytocannabinoids and other bioactive constituents of *Cannabis sativa*, particularly through well-designed human studies capable of determining the clinical relevance of these interactions, which will be discussed in the following sections.

## 4 Pharmacokinetic characteristics of phytocannabinoids

### 4.1 Absorption and routes of administration

Understanding the PK profile of phytocannabinoids is fundamental for predicting their therapeutic efficacy, safety, and potential for clinically relevant drug-drug interactions. Since many interactions involving cannabinoid-based therapies are pharmacokinetic in nature, knowledge of the absorption, distribution, metabolism, and elimination of these compounds is essential for anticipating changes in systemic drug exposure and therapeutic response. A comparative summary of the principal PK characteristics of the phytocannabinoids discussed in this review is presented in Table 1. The PK behavior of phytocannabinoids is influenced by multiple factors, including their chemical structure, lipophilicity, plasma protein binding, metabolic pathways, formulation, and route of administration, resulting in important differences among compounds (Huestis, 2007; Lacerda et al., 2025).

**TABLE 1:** Comparative pharmacokinetic profile of phytocannabinoids discussed in this review.

Phytocannabinoid	Oral bioavailability	Tmax	Main metabolic enzymes	Terminal half-life	Main route of excretion	Clinical pharmacokinetic considerations	Key references
Δ^9^-Tetrahydrocannabinol (THC)	Low and variable (∼4–12%)	1–5 h (oral); 3–10 min (inhalation)	CYP2C9, CYP3A4	∼25–36 h (single use); >4 days in chronic users	Mainly feces; urinary metabolites	Formation of the active metabolite 11-hydroxy-THC; prolonged elimination in chronic users due to adipose tissue accumulation	(Grotenhermen, 2003; Huestis, 2007; Ujváry and Hanuš, 2016)
Cannabidiol (CBD)	Low and highly variable (∼6–19%); markedly increased with high-fat meals	2–5 h (oral); minutes after inhalation	CYP3A4, CYP2C19; UGT1A7, UGT1A9, UGT2B7	∼18–35 h (single dose); 2–5 days after repeated oral administration	Primarily feces; minor urinary excretion	Extensive first-pass metabolism; pronounced food effect; clinically relevant inhibition of CYP enzymes; marked interindividual variability	(Birnbaum et al., 2019; Millar et al., 2019; O’Sullivan et al., 2024; Na and Lee, 2025)
Cannabigerol (CBG)	Human data remain limited; likely low oral bioavailability	Limited human data	CYP2J2, CYP2C8, CYP2C9, CYP2D6, CYP3A4	Unknown	Unknown	Limited clinical evidence; potential CYP-mediated drug interactions; additional human pharmacokinetic studies are needed	(Treyer et al., 2023; Nachnani et al., 2024; Lacerda et al., 2025)
Cannabinol (CBN)	Limited human data	Limited human data	Likely CYP2C9 and CYP3A4	Limited clinical evidence	Presumed fecal and urinary excretion	Human pharmacokinetic data remain scarce; potential for pharmacodynamic interactions with CNS depressants	(Huestis, 2007; Nachnani et al., 2024)
Tetrahydrocannabivarin (THCV)	Limited human data	Limited human data	Presumed CYP-mediated metabolism	Unknown	Unknown	Pharmacokinetic profile remains poorly characterized; clinical significance of potential drug interactions has yet to be established	(Abioye et al., 2020; Lacerda et al., 2025)

The table summarizes the current evidence regarding oral bioavailability, time to maximum plasma concentration (Tmax), major metabolic enzymes, terminal elimination half-life, primary routes of excretion, and clinically relevant pharmacokinetic considerations for the principal phytocannabinoids addressed in this review. Values should be interpreted as approximate ranges because they may vary according to formulation, route of administration, dose, fed or fasting state, study design, and patient characteristics. Abbreviations: CBD, cannabidiol; THC, Δ9-tetrahydrocannabinol; CBG, cannabigerol; CBN, cannabinol; THCV, tetrahydrocannabivarin; Tmax, time to maximum plasma concentration; CYP, cytochrome P450; UGT, uridine 5′-diphospho-glucuronosyltransferase.

The absorption of major phytocannabinoids, particularly THC and CBD, occurs primarily through oral, inhalational, and oromucosal (commonly referred to as sublingual) administration, with each route exhibiting distinct PK characteristics (Figure 1). Oral administration is associated with relatively low and highly variable bioavailability, generally ranging from approximately 6%–20%, mainly because of poor aqueous solubility and extensive first-pass hepatic metabolism (McGilveray, 2005; Huestis, 2007). In contrast, inhalation provides rapid pulmonary absorption, producing higher bioavailability and a faster onset of action, making this route particularly suitable when immediate therapeutic effects are desired (Chayasirisobhon, 2021).

**FIGURE 1:**
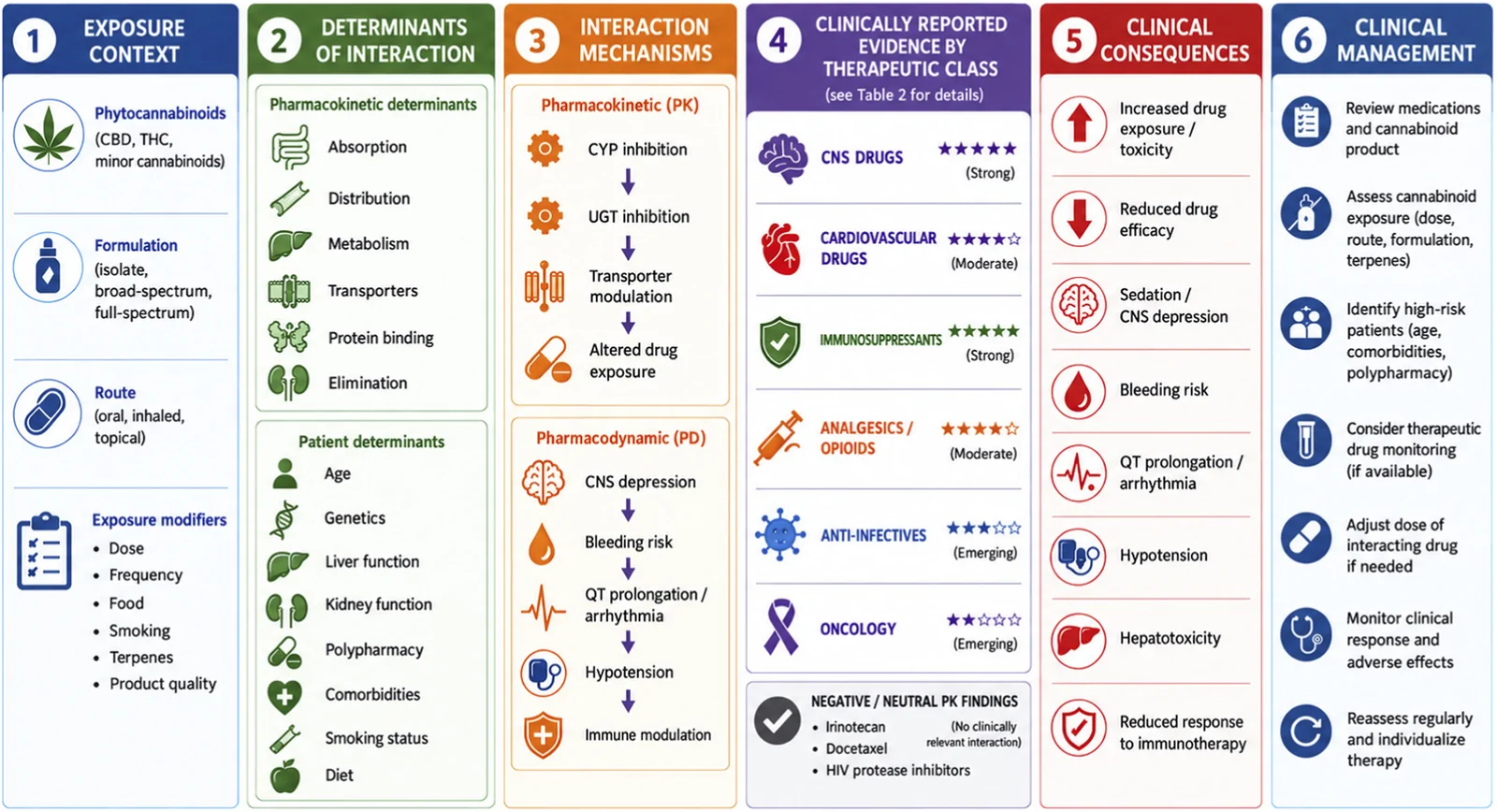
Clinical evidence map of phytocannabinoid–drug interactions. The figure summarizes the principal determinants, mechanisms, reported clinical evidence, consequences, and management strategies relevant to phytocannabinoid–drug interactions. (1) Exposure context includes phytocannabinoid composition, formulation, route of administration, and exposure modifiers that influence systemic cannabinoid exposure. (2) Determinants of interaction comprise pharmacokinetic characteristics (absorption, distribution, metabolism, transport, protein binding, and elimination) together with patient-specific factors such as age, genetics, hepatic and renal function, polypharmacy, comorbidities, smoking status, and diet. (3) Interaction mechanisms include pharmacokinetic mechanisms (enzyme and transporter modulation) and pharmacodynamic mechanisms (additive or synergistic pharmacological effects). (4) Therapeutic classes are presented according to the available evidence supporting phytocannabinoid–drug interactions, with star ratings and descriptors indicating the relative directness and extent of clinical support for the interactions summarized within each class. These indicators at the therapeutic-class level are intended to facilitate interpretation within Panel four and do not represent a universal hierarchy of study designs. Examples of reported neutral or non-clinically relevant pharmacokinetic interactions are also shown. (5) Potential clinical consequences include altered drug exposure or efficacy, adverse drug reactions, and treatment-related toxicities. (6) Clinical management emphasizes individualized risk assessment, medication review, therapeutic drug monitoring where appropriate, dose adjustment, and ongoing clinical follow-up. Abbreviations: CBD, cannabidiol; CNS, central nervous system; CYP, cytochrome P450; PD, pharmacodynamic; PK, pharmacokinetic; THC, Δ9-tetrahydrocannabinol; UGT, uridine 5′-diphospho-glucuronosyltransferase.

An additional consideration is that phytocannabinoids are biosynthesized predominantly in their acidic forms, including cannabidiolic acid (CBDA), Δ9-tetrahydrocannabinolic acid (THCA), and cannabigerolic acid (CBGA), which are the major constituents of raw Cannabis plant material. These acidic precursors undergo decarboxylation following exposure to heat, light, or prolonged storage, yielding their corresponding neutral cannabinoids. Consequently, formulations prepared from raw or cold-extracted Cannabis may retain substantially higher concentrations of acidic phytocannabinoids than smoked, vaporized, or thermally processed products. As these acidic cannabinoids exhibit distinct PK and PD properties and are increasingly present in commercially available formulations, they should also be considered when evaluating cannabinoid disposition and their potential contribution to pharmacokinetic and PK drug interactions (Anderson et al., 2019).

Although oromucosal administration has traditionally been considered capable of partially bypassing first-pass hepatic metabolism, recent PK evidence challenges this assumption. Studies evaluating CBD formulations have demonstrated PK parameters comparable to those observed after oral administration, suggesting that a substantial proportion of the administered dose is ultimately swallowed and absorbed through the gastrointestinal tract rather than directly through the oral mucosa (Johnson et al., 2024). These findings indicate that the PK advantages historically attributed to sublingual administration may have been overestimated for certain cannabinoid formulations.

In addition to the route of administration, several physiological and formulation-related factors can substantially influence cannabinoid bioavailability (Liu et al., 2018). Differences in physicochemical properties, lipid solubility, pharmaceutical formulation, and individual metabolic characteristics contribute to considerable interindividual variability in cannabinoid absorption and systemic exposure (Maldonado et al., 2024; Lacerda et al., 2025).

Food intake represents another clinically relevant determinant of cannabinoid PK. Oral administration of CBD with a high-fat meal has been shown to increase systemic exposure markedly, producing approximately a fourfold increase in the area under the concentration-time curve (AUC) and up to a fourteenfold increase in maximum plasma concentration (C_max) compared with fasting conditions (Birnbaum et al., 2019; O’Sullivan et al., 2024). These findings underscore the importance of maintaining consistency regarding food intake during oral cannabinoid therapy to reduce PK variability and optimize therapeutic outcomes (MacCallum and Russo, 2018). Collectively, these PK characteristics should be carefully considered during dose titration, therapeutic monitoring, and the evaluation of potential drug-drug interactions involving cannabinoid-based therapies.

### 4.2 Distribution

Following absorption, phytocannabinoids are rapidly distributed throughout the body because of their marked lipophilicity. Both THC and CBD readily partition into highly perfused tissues, including the brain, lungs, liver, and heart, before progressively accumulating in adipose tissue. This extensive tissue distribution contributes to their large apparent volume of distribution, prolonged terminal elimination phase, and considerable interindividual variability in PK profiles (Huestis, 2007; Chayasirisobhon, 2021).

An important PK characteristic of phytocannabinoids is their extensive binding to plasma proteins, particularly albumin and lipoproteins, with binding rates generally exceeding 95%. Consequently, alterations in plasma protein concentrations or competition with other highly protein-bound drugs may theoretically modify the free fraction of cannabinoids or co-administered medications. Although the clinical relevance of displacement interactions appears to be limited compared with metabolic interactions, these mechanisms may become more relevant in patients receiving multiple highly protein-bound medications or presenting conditions associated with hypoalbuminemia (Huestis, 2007; Smith and Gruber, 2023).

The high lipophilicity of phytocannabinoids also enables efficient penetration across biological barriers, including the blood-brain barrier, placenta, and mammary gland. Consequently, cannabinoids may accumulate within the CNS and reach fetal and neonatal tissues during pregnancy and lactation. These PK characteristics have important implications for efficacy, safety, and toxicity, particularly in vulnerable populations, reinforcing the need for careful risk-benefit assessment when cannabinoid-based therapies are prescribed during pregnancy, breastfeeding, or in other special clinical situations (Grant et al., 2018; Navarrete et al., 2020).

### 4.3 Metabolism

The metabolism of phytocannabinoids occurs predominantly in the liver and represents the principal mechanism underlying many clinically relevant drug-drug interactions involving cannabinoid-based therapies. Following absorption, cannabinoids undergo extensive biotransformation through Phase I and Phase II metabolic pathways, producing metabolites with distinct pharmacological and toxicological properties. Interindividual variability in these metabolic processes may substantially influence systemic drug exposure, therapeutic efficacy, and the occurrence of adverse drug reactions (Huestis, 2007; Bardhi et al., 2022).

Phase I metabolism is mediated primarily by cytochrome P450 (CYP450) enzymes, particularly CYP2C9, CYP2C19, and CYP3A4. THC is metabolized mainly by CYP2C9 and CYP3A4 to form 11-hydroxy-THC (11-OH-THC), an active metabolite that contributes significantly to the psychoactive effects of *Cannabis*, before being further oxidized to the inactive metabolite 11-nor-9-carboxy-THC (THC-COOH). In contrast, CBD is metabolized predominantly by CYP2C19 and CYP3A4, generating metabolites such as 7-hydroxy-CBD (7-OH-CBD), which retains pharmacological activity, and 7-carboxy-CBD (7-COOH-CBD), the major inactive circulating metabolite (Ujváry and Hanuš, 2016; Beers et al., 2021).

Following Phase I metabolism, cannabinoids undergo Phase II conjugation reactions, primarily mediated by UGTenzymes, especially UGT1A9 and UGT2B7, facilitating renal and biliary elimination. Although Phase II metabolism has received less attention than CYP-mediated pathways, increasing evidence indicates that alterations in UGT activity may also contribute to clinically relevant PK interactions (Bansal et al., 2020; Smith and Gruber, 2023).

Beyond acting as metabolic substrates, phytocannabinoids may also function as inhibitors of drug-metabolizing enzymes. CBD, in particular, has been shown to inhibit several CYP isoforms, including CYP2C19, CYP2C9, CYP2D6, and CYP3A4, whereas THC generally exhibits weaker inhibitory effects. Consequently, concomitant administration of cannabinoid-based therapies with medications primarily metabolized by these enzymes may increase systemic drug exposure and the risk of adverse effects. Conversely, genetic polymorphisms affecting CYP2C9 and CYP2C19 activity may alter cannabinoid metabolism, contributing to substantial interindividual variability in treatment response and safety (Bansal et al., 2020; Bardhi et al., 2022).

Understanding these metabolic pathways is essential for predicting clinically relevant drug interactions and provides the mechanistic basis for many of the PK interactions discussed in the following sections.

### 4.4 Elimination

Following hepatic metabolism, phytocannabinoids are eliminated primarily as polar metabolites through urinary and fecal excretion. Renal elimination occurs predominantly after Phase II conjugation, whereas biliary excretion contributes substantially to the elimination of several cannabinoid metabolites (Ujváry and Hanuš, 2016). The elimination profile of phytocannabinoids is strongly influenced by their pronounced lipophilicity, which promotes extensive distribution into adipose tissue followed by gradual redistribution into the systemic circulation. Consequently, the prolonged terminal elimination observed for cannabinoids reflects not only metabolic clearance but also the slow release of these compounds from peripheral tissue reservoirs (Huestis, 2007).

Among phytocannabinoids, THC exhibits the longest elimination profile because of its extensive tissue accumulation. In chronic users, the terminal plasma half-life may exceed 4 days, while its principal inactive metabolite, 11-nor-9-carboxy-THC (THC-COOH), presents an elimination half-life of approximately five to 6 days (Huestis, 2007). Importantly, prolonged elimination should not be interpreted as prolonged pharmacological activity, since psychoactive effects generally resolve long before complete systemic elimination.

CBD also exhibits route-dependent elimination kinetics. Reported elimination half-lives are approximately 24 h following intravenous administration, around 31 h after inhalation, and between two and 5 days after repeated oral administration (Millar et al., 2019). In addition to the route of administration, cannabinoid elimination is influenced by treatment duration, adipose tissue accumulation, hepatic metabolic capacity, renal excretion, and interindividual variability, resulting in considerable differences in systemic exposure and drug persistence among patients (Huestis, 2007; Millar et al., 2019).

The major urinary metabolites, including THC-COOH and 7-carboxy-cannabidiol (7-COOH-CBD), provide valuable biomarkers of cannabinoid exposure and are widely employed in clinical and forensic toxicology (Alsherbiny and Li, 2018). However, the detection of these metabolites primarily reflects previous cannabinoid exposure and should not be interpreted as evidence of current pharmacological impairment or acute intoxication. Therefore, appropriate interpretation of cannabinoid elimination kinetics is essential not only for therapeutic monitoring but also for toxicological assessment and the interpretation of biological testing.

Collectively, these PK characteristics provide the mechanistic basis for understanding the complex drug-drug interactions associated with phytocannabinoids, which are discussed in the following section.

## 5 Pharmacokinetic and pharmacodynamic drug interactions

Drug-drug interactions involving phytocannabinoids have become an increasingly important clinical concern as cannabinoid-based therapies are progressively incorporated into the management of neurological, psychiatric, inflammatory, and chronic pain conditions. An integrated overview of the factors, mechanisms, reported interactions, and clinical implications addressed in this review is provided in Figure 1. The principal clinically relevant drug interactions discussed throughout this review are summarized in Table 2. The risk of clinically relevant interactions is particularly pronounced among patients receiving polypharmacy, including antidepressants, antiepileptic drugs, anticoagulants, immunosuppressants, and cardiovascular medications (Crippa et al., 2018; Amaral Silva et al., 2020; Papakyriakopoulou et al., 2026). Most cannabinoid-related drug interactions are pharmacokinetic in nature and result primarily from inhibition or, less frequently, induction of drug-metabolizing enzymes, particularly members of the cytochrome P450 family. However, PD interactions resulting from additive or synergistic effects on the CNS or other physiological pathways also contribute substantially to the clinical profile of cannabinoid-based therapies (Maldonado et al., 2024; Caicedo et al., 2025).

**TABLE 2:** Clinically relevant drug interactions involving phytocannabinoids**.**

Phytocannabinoid	Drug/class involved	Proposed mechanism	Possible clinical consequence	Clinical recommendation	Evidence level	Key references
CBD	Clobazam and other benzodiazepines	Inhibition of CYP2C19 and CYP3A4; increased exposure to N-desmethylclobazam; additive CNS depression	Somnolence, excessive sedation, dizziness, psychomotor impairment	Monitor sedation; consider dose reduction of clobazam or benzodiazepine; use caution in older adults and polypharmacy	Controlled clinical evidence	(Cox et al., 2019; Bansal et al., 2020; Nachnani et al., 2024)
	Valproate	Pharmacodynamic interaction and possible hepatic vulnerability; mechanism not fully established	Increased hepatic transaminases; potential hepatotoxicity	Monitor liver function tests, especially during initiation and dose escalation	Prospective observational studies	(Silvestro et al., 2019; Na and Lee, 2025)
	Warfarin and coumarinic anticoagulants	CYP2C9 inhibition; possible CYP3A4 involvement	Increased INR; increased bleeding risk	Monitor INR closely; consider anticoagulant dose adjustment when CBD is initiated, discontinued, or titrated	Case reports/case series	(Alsherbiny and Li, 2018; Balachandran et al., 2021; Treyer et al., 2023)
	Antidepressants, including SSRIs and tricyclic antidepressants	Inhibition of CYP2C19, CYP2D6, and CYP3A4	Increased antidepressant exposure; sedation, gastrointestinal adverse effects, QT-related concerns for susceptible drugs	Review metabolic pathways; monitor adverse effects; adjust dose when clinically indicated	Human PK interaction studies	(Arellano et al., 2017; Cox et al., 2019; Campos et al., 2024)
	Antipsychotics	CYP2D6 and CYP3A4 modulation; additive CNS effects	Sedation, orthostatic hypotension, cognitive impairment; altered antipsychotic exposure	Monitor CNS adverse effects and cardiovascular tolerability; individualize dose adjustments	Prospective observational studies	(Boggs et al., 2018; Crippa et al., 2018; Campos et al., 2024)
	Immunosuppressants, especially tacrolimus and cyclosporine	CYP3A4 and P-gp inhibition	Increased immunosuppressant exposure; nephrotoxicity or systemic toxicity	Therapeutic drug monitoring is recommended; coordinate with prescribing specialist	Human PK interaction studies	(Balachandran et al., 2021; Caicedo et al., 2025; Papakyriakopoulou et al., 2026)
CBD and cannabinoid-based products	Antihypertensive medications	Pharmacodynamic additivity through reduced vascular resistance, vasorelaxation, altered autonomic regulation, and possible reduction in arterial stiffness; potential CYP3A4/CYP2D6-mediated interaction for selected drugs remains theoretical	Excessive blood pressure reduction, orthostatic hypotension, dizziness, falls, or syncope; antihypertensive dose reduction may occasionally be required	Monitor seated and standing blood pressure during treatment initiation and titration; use particular caution in older adults, patients receiving multiple antihypertensives, and those using diuretics; adjust antihypertensive therapy according to clinical response	Prospective observational studies	(Jadoon et al., 2017; Abuhasira et al., 2021; Balachandran et al., 2021; Watanabe et al., 2021; Dragun et al., 2023)
THC	Alcohol	Additive CNS impairment; alcohol may increase THC exposure after inhalation	Greater impairment of attention, reaction time, motor coordination, and driving performance	Avoid concomitant use; counsel patients regarding driving and safety-sensitive activities	Controlled clinical studies	(Lukas and Orozco, 2001; Hartman et al., 2015)
	Benzodiazepines and other CNS depressants	Additive CNS depression	Sedation, dizziness, falls, cognitive and psychomotor impairment	Use caution; avoid high THC doses; monitor closely in older adults and patients using multiple CNS depressants	Prospective observational studies	(Boggs et al., 2018; Balachandran et al., 2021; Nachnani et al., 2024)
	Opioids	Pharmacodynamic interaction involving cannabinoid and opioid systems; possible opioid-sparing effect but additive CNS adverse effects	Enhanced analgesia; sedation, dizziness, cognitive impairment; potential safety concerns	Consider lower opioid doses when appropriate; monitor sedation, cognition, and respiratory risk factors	Controlled clinical studies	(Desroches and Beaulieu, 2010; Scavone et al., 2013; Nielsen et al., 2017)
	CYP2C9/CYP3A4 inhibitors, including ketoconazole, fluconazole, amiodarone, and trimethoprim-sulfamethoxazole	Reduced THC metabolism through CYP2C9 and/or CYP3A4 inhibition	Increased THC exposure; prolonged psychoactive effects; anxiety, tachycardia, dizziness, cognitive impairment	Use caution; consider dose reduction of THC-containing products; monitor adverse effects	Human PK interaction studies	(Antoniou et al., 2020; Treyer et al., 2023)
THC-containing cannabis products	Immune checkpoint inhibitors, particularly PD-1/PD-L1 inhibitors	CB2/CNR2-mediated suppression of T-cell activation and inhibition of JAK1–STAT1/STAT3 signaling; evidence is primarily preclinical, with limited observational clinical data	Possible reduction in tumor response or immunotherapy efficacy; effects on progression-free and overall survival remain uncertain	Assess cannabinoid use before and during immunotherapy; discuss with the oncology team; use caution, especially with THC-rich products; monitor tumor response closely	Prospective observational studies	(Taha et al., 2019; Xiong et al., 2022; Vigano et al., 2025)
CBG	Warfarin and other CYP-metabolized drugs	Potential CYP-mediated inhibition; limited clinical evidence	Possible increased drug exposure; uncertain clinical magnitude	Use caution with narrow therapeutic index drugs; monitor clinically and consider laboratory monitoring when appropriate	Preclinical/PBPK/*in vitro* studies	(Treyer et al., 2023; Lacerda et al., 2025)
CBN	Benzodiazepines and CNS depressants	Potential additive sedative effects; limited pharmacokinetic evidence	Sedation, dizziness, psychomotor impairment	Avoid combining with multiple sedatives when possible; start with low doses and monitor CNS effects	Preclinical/PBPK/*in vitro* studies	(Babson et al., 2017; Balachandran et al., 2021)
THCV	Antiepileptics, antidepressants, and antipsychotics	Poorly characterized; presumed CYP-mediated interaction potential	Unknown clinical relevance; potential changes in exposure or adverse effects	Evidence is insufficient; use caution and monitor when co-administered with narrow therapeutic index or CNS-active drugs	Preclinical/PBPK/*in vitro* studies	(Abioye et al., 2020; Lacerda et al., 2025)

The table summarizes drug classes and medications with potential or documented interactions with phytocannabinoids, highlighting the proposed mechanisms, possible clinical consequences, practical recommendations for clinical monitoring and risk reduction, and the corresponding Evidence level, assigned according to the evidence-classification framework presented in Figure 2. When multiple types of evidence were available, the classification reflects the highest level of evidence currently supporting the interaction. This classification is intended to facilitate differentiation between clinically established interactions and those supported by lower levels of clinical or preclinical evidence. The clinical relevance of each interaction may vary according to cannabinoid formulation, dose, route of administration, treatment duration, patient-specific factors, and concomitant pharmacotherapy.

The clinical relevance of these interactions is likely to vary according to cannabinoid composition, dose, route of administration, treatment duration, and patient-specific characteristics, including age, genetic polymorphisms, hepatic function, and concomitant medications (Chen et al., 2024; Caicedo et al., 2025). Consequently, individualized therapeutic assessment remains essential to maximize treatment efficacy while minimizing adverse drug reactions. Accordingly, both the discussion throughout this review and the classification presented in Table 2 distinguish interactions supported by human clinical evidence from those based primarily on shared metabolic pathways, *in vitro* findings, or preclinical studies, thereby facilitating interpretation of their respective evidential basis and clinical relevance.

### 5.1 Pharmacokinetic drug interactions

Most currently documented cannabinoid-related drug interactions are PK in nature and result primarily from inhibition or, less frequently, induction of drug-metabolizing enzymes, particularly cytochrome P450 isoforms. This predominance reflects both the ability of phytocannabinoids—particularly CBD—to inhibit multiple drug-metabolizing enzymes and the greater availability of measurable PK endpoints, such as plasma concentrations, AUC, Cmax, and metabolite exposure. However, the predominance of PK evidence should not be interpreted as indicating that PD interactions are necessarily less clinically important, as these are often more difficult to quantify and may remain underrecognized**.** These interactions occur predominantly through modulation of drug-metabolizing enzymes and, to a lesser extent, membrane transporters, resulting in altered plasma concentrations, therapeutic responses, and adverse effect profiles of co-administered medications. Among phytocannabinoids, CBD has been consistently identified as the compound with the greatest potential to precipitate clinically relevant PK interactions because of its inhibitory effects on multiple cytochrome P450 (CYP450) and UGT enzymes (Bansal et al., 2020; Smith and Gruber, 2023; Caicedo et al., 2025).

Beyond the inhibitory properties described above, accumulating evidence indicates that CBD may also produce time-dependent inhibition of CYP3A4 and CYP2C19, further increasing its interaction potential during repeated administration (Bansal et al., 2020). Consequently, concomitant use of CBD with drugs metabolized through these pathways may increase systemic drug exposure and the risk of concentration-dependent adverse effects. Non-CYP mechanisms may also contribute to phytocannabinoid-related PK interactions. *In vitro* studies indicate that CBD, THC, and several of their metabolites can inhibit multiple UGT enzymes, including UGT1A6, UGT1A9, UGT2B4, and UGT2B7, although the magnitude of inhibition varies across cannabinoids and substrates (Nasrin et al., 2021). CBD and THC have also been shown to inhibit UGT2B7-mediated morphine glucuronidation in human liver microsomes, providing a mechanistic basis for a potential interaction with morphine; however, this interaction has not yet been confirmed in controlled clinical co-administration studies (Coates et al., 2024). Phytocannabinoids may additionally modulate ATP-binding cassette transporters, including P-glycoprotein, breast cancer resistance protein, and multidrug resistance-associated protein 1, potentially affecting drug absorption, tissue distribution, and elimination (Zhu et al., 2006; Holland et al., 2007; 2008). Nevertheless, transporter findings remain predominantly *in vitro*, are sometimes substrate- and model-dependent, and should therefore be regarded as mechanistically plausible rather than clinically established interactions.

Direct human evidence of CBD-mediated CYP inhibition was provided by a randomized crossover study in 18 healthy adults that evaluated a CBD-dominant *Cannabis* extract (640 mg CBD +20 mg THC) using caffeine, losartan, omeprazole, dextromethorphan, and midazolam as CYP probe substrates. Compared with placebo, the CBD-dominant extract increased the AUC of omeprazole (CYP2C19) by 207%, losartan (CYP2C9) by 77%, midazolam (CYP3A) by 56%, and caffeine (CYP1A2) by 39%, while producing no meaningful inhibition of CYP2D6. Importantly, the THC-dominant extract did not inhibit any of the evaluated CYP pathways, whereas CBD co-administration increased Δ9-THC exposure (AUC) by 161%, providing direct human evidence for both CBD–drug interactions and an intraproduct CBD–THC PK interaction (Bansal et al., 2023). Consistent with these findings, a separate Phase I fixed-sequence study showed that repeated administration of high-dose CBD increased caffeine AUC by 95% and prolonged its elimination half-life from 5.4 to 10.9 h, providing further controlled clinical evidence of CYP1A2 inhibition during steady-state CBD exposure (Thai et al., 2021). However, the relevance of this interaction at the substantially lower CBD doses commonly found in nonprescription consumer products remains uncertain.

Among the best-characterized clinically relevant cannabinoid-drug interactions, the combination of CBD with the benzodiazepine antiseizure medication clobazam has been extensively documented (Geffrey et al., 2015; Gaston et al., 2017; Morrison et al., 2019). CBD inhibits CYP2C19-mediated metabolism of N-desmethylclobazam, the active metabolite of clobazam, leading to substantial increases in plasma concentrations and a higher incidence of excessive sedation and somnolence. A potentially relevant interaction has also been reported with brivaracetam. In a case series of five patients receiving stable brivaracetam therapy, the introduction of CBD was associated with increases in brivaracetam plasma concentrations ranging from 95% to 280%. Two patients experienced mild potentially related adverse effects, and brivaracetam dose reduction was required in one case. Although inhibition of CYP2C19 may contribute to this interaction, the evidence remains limited to a small case series and requires confirmation in controlled PK studies (Klotz et al., 2019).

Beyond clobazam, CBD-associated changes have been observed in the circulating concentrations of several antiseizure medications, including topiramate, rufinamide, zonisamide, eslicarbazepine, and stiripentol (Gilmartin et al., 2021). However, the magnitude and clinical relevance of these changes are generally less consistent than those observed for N-desmethylclobazam. Concomitant valproate use is particularly relevant from a toxicological perspective because it is associated with a greater risk of hepatic transaminase elevations during CBD treatment, despite the absence of a consistently demonstrated increase in valproate exposure (Chen et al., 2024). This interaction may therefore be more appropriately regarded as PD or toxicodynamic than as a conventional PK interaction.

Enzyme-mediated interactions have additionally been reported or predicted for medications belonging to several therapeutic classes, including antidepressants, antipsychotics, other benzodiazepines, and cardiovascular agents metabolized by CYP3A4 and CYP2C19 (Arellano et al., 2017; Cox et al., 2019; Nachnani et al., 2024). Among antidepressants, an interaction between CBD and citalopram or escitalopram has been supported by both mechanistic and preliminary clinical evidence. CBD inhibited citalopram and escitalopram metabolism *in vitro*, and its addition to stable treatment was associated with increased antidepressant plasma concentrations in a small clinical cohort. Although the clinical consequences remain incompletely defined, it remains uncertain whether these PK changes translate into a clinically meaningful increase in SSRI-related adverse effects. Nevertheless, monitoring for concentration-dependent adverse effects may be warranted, particularly during CBD initiation or dose escalation (Anderson et al., 2021). The effects of *Cannabis* smoking should be distinguished from those of orally or oromucosally administered phytocannabinoids. Polycyclic aromatic hydrocarbons generated during combustion can induce CYP1A2, potentially increasing the clearance of CYP1A2 substrates. Conversely, reduction or cessation of *Cannabis* smoking may reverse this induction and increase exposure to drugs such as clozapine and olanzapine, with reported cases of concentration-related toxicity (Zullino et al., 2002). This route-specific interaction is attributable primarily to smoke exposure rather than to phytocannabinoids themselves and should not be extrapolated to non-combusted formulations.

Clinically important PK interactions have also been demonstrated between CBD and immunosuppressants, particularly calcineurin inhibitors and mechanistic target of rapamycin (mTOR) inhibitors. In a controlled Phase I study, steady-state CBD increased the maximum concentration (Cmax) and area under the concentration–time curve (AUC) of tacrolimus by 4.2-fold and 3.1-fold, respectively (So et al., 2025). A separate Phase I study demonstrated an approximately 2.5-fold increase in both the Cmax and AUC of everolimus during co-administration with CBD (Wray et al., 2023). These controlled findings are further supported by real-world clinical observations. In a retrospective clinical series of patients receiving everolimus or sirolimus, trough concentrations increased after CBD initiation in 76% of patients, with some experiencing a doubling or tripling of mTOR inhibitor concentrations. Clinical toxicity and laboratory abnormalities were observed in some patients, prompting dose adjustments and closer therapeutic drug monitoring (Ebrahimi-Fakhari et al., 2020). Case-level evidence has additionally described a PK interaction between CBD and the immunosuppressants cyclosporine and mycophenolate mofetil in a kidney-transplant recipient, potentially involving inhibition of CYP3A-mediated cyclosporine metabolism and carboxylesterase-dependent mycophenolate disposition; however, the magnitude and generalizability of this interaction remain uncertain (Cuñetti et al., 2024). Given the narrow therapeutic indices of these immunosuppressants, CBD initiation, discontinuation, or dose modification may require intensified therapeutic drug monitoring and appropriate adjustment of immunosuppressant dosing.

PK interactions may also contribute to the effects of cannabinoid–opioid combinations. A clinically significant CBD–methadone interaction has been reported, with increased methadone exposure accompanied by excessive sedation, while an observational study in patients receiving opioid-maintenance treatment found substantially higher buprenorphine concentrations among concomitant *Cannabis* users (Madden et al., 2020; Vierke et al., 2021). Although these findings are less definitive than the CBD–clobazam and CBD–tacrolimus interactions and are based on limited clinical evidence, they suggest that patients receiving methadone or buprenorphine may require closer monitoring when cannabinoid use is initiated, discontinued, or substantially modified.

Unlike CBD, THC generally exhibits a lower potential for metabolic interactions, although clinically relevant effects may still occur. THC is metabolized primarily by CYP2C9 and CYP3A4, and co-administration with inhibitors of these enzymes, including ketoconazole, fluconazole, amiodarone, and trimethoprim-sulfamethoxazole, may increase systemic THC exposure, prolong psychoactive effects, and enhance the risk of adverse reactions (Antoniou et al., 2020; Treyer et al., 2023). Conversely, enzyme induction may accelerate THC metabolism, reducing therapeutic efficacy. Phytocannabinoids may also act as victims of PK interactions. In healthy volunteers receiving a THC/CBD oromucosal formulation, the strong CYP3A inhibitor ketoconazole increased THC, CBD, and 11-hydroxy-THC exposure, whereas the enzyme inducer rifampicin substantially reduced exposure to both phytocannabinoids and their metabolites. These findings indicate that initiation or discontinuation of strong CYP inhibitors or inducers may alter cannabinoid efficacy and tolerability and may necessitate renewed dose titration (Stott et al., 2013).

Although clinical evidence remains considerably more limited for minor phytocannabinoids, available *in vitro* studies indicate that compounds such as CBG and CBN also interact with hepatic drug-metabolizing enzymes. Oxidative metabolism of CBG involves CYP2J2, CYP3A4, CYP2D6, CYP2C8, and CYP2C9, whereas *in vitro* studies using human liver microsomes and recombinant human CYP enzymes have demonstrated varying degrees of inhibition of CYP2A6 and CYP2B6 by THC, CBD, and CBN (Matsunaga et al., 2000; Treyer et al., 2023). Taken together, these findings support mechanistic plausibility but do not establish the magnitude, direction, or clinical consequences of CBG- or CBN-mediated interactions in humans. The increasing use of full-spectrum *Cannabis* preparations therefore highlights the need to better characterize the PK profiles and interaction potential of minor phytocannabinoids.

Particular attention should be given to medications with a narrow therapeutic index. Anticoagulants such as warfarin warrant particular caution, as inhibition of CYP2C9 by CBD and other phytocannabinoids may increase warfarin drug concentrations, elevate the international normalized ratio (INR), and substantially increase the risk of bleeding (Alsherbiny and Li, 2018; Caicedo et al., 2025). For warfarin specifically, however, the available clinical evidence remains largely case-based rather than derived from controlled cannabinoid–warfarin co-administration studies. Similar caution is warranted for immunosuppressants, certain antiarrhythmics, and other drugs for which relatively small changes in systemic exposure may result in clinically significant toxicity. Therefore, careful dose adjustment, therapeutic drug monitoring when appropriate, and individualized clinical assessment remain essential during cannabinoid-based therapy.

Importantly, shared metabolic pathways and inhibitory effects observed *in vitro* do not invariably translate into clinically meaningful PK interactions. In patients with cancer, administration of standardized medicinal *Cannabis* as an herbal tea for 15 consecutive days did not significantly alter the systemic exposure or clearance of irinotecan, its active metabolite, or docetaxel, despite the involvement of CYP3A-dependent pathways in the disposition of these anticancer agents (Engels et al., 2007). Similarly, in a randomized placebo-controlled study of patients with HIV receiving indinavir or nelfinavir, short-term administration of smoked *Cannabis* or oral dronabinol produced only modest changes in protease-inhibitor PK that were considered unlikely to have clinically important short-term consequences (Kosel et al., 2002). Furthermore, in patients with chronic pain receiving stable doses of morphine or oxycodone, the addition of vaporized *Cannabis* enhanced analgesia without significantly altering plasma opioid concentrations, suggesting that the observed cannabinoid–opioid interaction was predominantly pharmacodynamic rather than pharmacokinetic (Abrams et al., 2011). Collectively, these findings demonstrate that mechanistic plausibility alone is insufficient to establish clinical relevance and reinforce the importance of considering cannabinoid formulation, dose, route of administration, duration of exposure, the specific co-administered medication, and the strength of the supporting human evidence when evaluating potential phytocannabinoid–drug interactions.

### 5.2 Pharmacodynamic drug interactions

In addition to PK interactions, phytocannabinoids may produce PD interactions through additive, synergistic, or antagonistic effects on shared physiological pathways. Unlike PK interactions, these effects can occur without changes in systemic drug concentrations and may involve depression, psychomotor impairment, analgesia, cardiovascular regulation, haemostasis, or immune function. However, direct controlled evidence remains limited for many proposed PD interactions, and mechanistic plausibility should not be interpreted as evidence of established clinical significance (Gottschling et al., 2020; Balachandran et al., 2021).

The most clinically apparent PD interactions involve additive CNS depression. THC-containing products may enhance sedation, dizziness, cognitive impairment, and psychomotor slowing when combined with benzodiazepines, sedative-hypnotics, antiseizure medications, antipsychotics, opioids, or other centrally acting depressants. These effects may increase the risk of impaired driving, falls, and accidental injury, particularly in older adults and patients receiving multiple centrally acting medications. CBD is generally less impairing than THC; however, somnolence and sedation may still occur, especially at higher therapeutic doses or when CBD increases exposure to a concomitant sedative medication.

The CBD–clobazam interaction illustrates the overlap between PK and PD mechanisms. CBD inhibits CYP2C19-mediated clearance of N-desmethylclobazam, increasing exposure to the active metabolite, while the resulting somnolence and sedation reflect both the PK increase in metabolite exposure and additive CNS-depressant effects. Although this is the best-characterized benzodiazepine-related interaction, direct controlled evidence for PD interactions with benzodiazepines other than clobazam remains limited. These risks should therefore be regarded as clinically plausible rather than firmly established, particularly in older adults and patients receiving multiple CNS depressants (Boggs et al., 2018; Nachnani et al., 2024).

Interactions between phytocannabinoids and opioids have attracted considerable interest because of their potential therapeutic implications for pain management. Experimental and clinical studies suggest that cannabinoids may enhance opioid-induced analgesia through complementary modulation of cannabinoid and opioid receptor systems, potentially allowing opioid dose reduction and contributing to opioid-sparing strategies in chronic pain management (Desroches and Beaulieu, 2010; Gottschling et al., 2020). In patients with chronic pain receiving stable doses of morphine or oxycodone, vaporized *Cannabis* enhanced analgesia without significantly altering plasma opioid concentrations, supporting a predominantly PD interaction (Abrams et al., 2011). Similarly, in a double-blind, placebo-controlled crossover study, smoked *Cannabis* combined with a subanalgesic dose of oxycodone increased pain threshold and tolerance when neither treatment produced significant analgesia alone, although the combination resulted in small increases in ratings associated with oxycodone abuse liability (Cooper et al., 2018). In a subsequent randomized crossover study, inhaled THC did not exacerbate oxycodone-induced respiratory depression in healthy volunteers, although a slight increase in sedation was observed (van Dam et al., 2023). Collectively, these findings support the possibility of analgesic synergy under controlled conditions but do not establish sustained opioid-sparing efficacy or long-term safety. Monitoring for sedation, cognitive impairment, and misuse-related effects therefore remains warranted during combined treatment. Nevertheless, additive adverse effects, including sedation, dizziness, impaired psychomotor performance, and cognitive impairment, should also be considered during combined therapy (Scavone et al., 2013; Nielsen et al., 2017).

The concomitant use of cannabinoids and alcohol represents one of the best-characterized cannabinoid-related PD interactions, as both substances independently impair cognitive and psychomotor performance. Controlled studies indicate that combined exposure produces greater impairment of attention, reaction time, motor coordination, and driving performance than either substance alone (Ronen et al., 2010; Downey et al., 2013). In addition, alcohol has been reported to increase plasma THC concentrations following inhalation, potentially enhancing its psychoactive effects and increasing the likelihood of adverse events (Lukas and Orozco, 2001; Hartman et al., 2015).

Potential PD interactions with psychostimulant medications also warrant consideration. In a controlled crossover study, co-administration of THC and methylphenidate produced additive increases in heart rate and rate–pressure product and altered subjective and performance-related effects compared with either drug alone (Kollins et al., 2015). Although this evidence derives from a small sample of healthy adults and cannot be generalized to all psychostimulants, it supports particular caution during cannabinoid co-administration in patients receiving methylphenidate, especially those with cardiovascular disease, baseline tachycardia, or other cardiovascular risk factors.

Potential PD additivity between cannabinoid-based therapies and antihypertensive medications also warrant clinical attention. Acute administration of CBD has been shown to reduce resting systolic blood pressure and attenuate blood pressure responses to stress in healthy volunteers, whereas ambulatory monitoring studies in individuals with untreated hypertension and older adults receiving medical *Cannabis* have demonstrated modest but significant reductions in systolic and diastolic blood pressure (Jadoon et al., 2017; Abuhasira et al., 2021; Dragun et al., 2023). In addition, randomized clinical evidence indicates an increased risk of hypotension and orthostatic hypotension during medical cannabinoid use (Watanabe et al., 2021). These effects may be clinically relevant in patients receiving antihypertensive therapy because additive blood pressure-lowering effects could increase the risk of symptomatic hypotension, orthostatic intolerance, dizziness, falls, and syncope (Watanabe et al., 2021; Fedorowski et al., 2022). However, direct controlled studies evaluating cannabinoid–antihypertensive combinations remain lacking, and this interaction should therefore be regarded as potential rather than clinically established. Blood pressure and orthostatic symptoms should be monitored during cannabinoid initiation and dose titration, particularly in older adults, individuals receiving multiple antihypertensive agents, and patients treated with diuretics or other medications associated with orthostatic hypotension.

Potential PD interactions with antipsychotic medications should be interpreted according to phytocannabinoid composition. Controlled human studies demonstrate that THC can transiently exacerbate psychotic and cognitive symptoms, potentially opposing the therapeutic objectives of antipsychotic treatment (D’Souza et al., 2004; 2005). CBD, by contrast, has been investigated as an adjunct to existing antipsychotic therapy and was generally well tolerated, with preliminary evidence of improvement in positive psychotic symptoms (McGuire et al., 2018). These divergent effects preclude treating cannabinoids as a homogeneous pharmacological class. Direct controlled evidence that cannabinoid–antipsychotic co-administration produces additive sedation, orthostatic hypotension, and cognitive impairment remains limited, and these risks should therefore be characterized as clinically plausible and dependent on cannabinoid composition, dose, and patient vulnerability. (Boggs et al., 2018; Crippa et al., 2018).

Emerging concern has also focused on potential interactions between cannabinoids and cancer immunotherapy. In an early retrospective study of patients with advanced malignancies receiving the programmed cell death protein 1 (PD-1) immune checkpoint inhibitor nivolumab, concomitant *Cannabis* use was associated with a lower tumor response rate, although no significant differences were observed in progression-free or overall survival (Taha et al., 2019). A subsequent retrospective cohort also associated cannabinoid use with poorer overall survival, progression-free survival, and disease control among patients receiving immune checkpoint inhibitors (Hadid et al., 2024). Conversely, a 2025 pooled individual-patient analysis of 684 participants from four clinical trials found that baseline cannabinoid use was associated with improved immune-related progression-free survival but not with conventional progression-free survival, overall survival, response, or immune-related adverse events; no evidence of a detrimental effect was observed during combination immune-checkpoint-inhibitor therapy (Coschi et al., 2025).

Preclinical studies provide biological plausibility for an antagonistic interaction, as THC has been shown to impair tumour-specific T-cell function and reduce the therapeutic effects of PD-1 blockade through CB2-dependent inhibition of JAK1–STAT1/STAT3 signaling (Xiong et al., 2022). Nevertheless, clinical evidence remains heterogeneous. Observational studies have suggested that concomitant cannabinoid use may reduce the incidence of immune-related adverse events; however, these findings have not been consistently accompanied by improvements in objective response rates, progression-free survival, or overall survival (Bar-Sela et al., 2013; Vigano et al., 2025). Therefore, concomitant cannabinoid use during immune checkpoint inhibitor therapy should be evaluated individually, considering cannabinoid composition, therapeutic indication, tumor characteristics, and the potential risk of reduced immunotherapy efficacy (Vigano et al., 2025).

Overall, phytocannabinoid-related PD interactions may produce beneficial therapeutic effects, clinically important adverse outcomes, or both, depending on cannabinoid composition, dose, concomitant medication, and patient-specific susceptibility. The strongest direct human evidence concerns combined use with alcohol and opioids (Lucas et al., 2018; Caicedo et al., 2025), whereas interactions involving psychostimulants remain supported primarily by mechanistic, observational, and limited controlled clinical evidence (Daldegan-Bueno et al., 2021; Daldegan-Bueno et al., 2022). Similarly, interactions with benzodiazepines, antihypertensive medications, antipsychotics, and immune checkpoint inhibitors are supported by varying combinations of mechanistic, observational, and limited clinical evidence (Xiong et al., 2022; Caicedo et al., 2025). Clear differentiation between established PD interactions and those based primarily on overlapping pharmacological effects is therefore essential for individualized risk assessment and safe cannabinoid–medication co-administration.

Importantly, clinically relevant cannabinoid-related drug interactions frequently arise from the interplay between PK alterations, PD mechanisms, and their toxicological consequences rather than from a single mechanism in isolation (Gottschling et al., 2020; Balachandran et al., 2021; Caicedo et al., 2025). For example, enzyme inhibition may increase systemic drug exposure, while concomitant PD effects such as enhanced sedation, hypotension, or immunomodulation may further amplify the likelihood and severity of adverse clinical outcomes. Therefore, an integrated evaluation of PK, PD, and toxicological evidence is essential for accurate risk assessment and individualized therapeutic decision-making (Smith and Gruber, 2023; Papakyriakopoulou et al., 2026).

### 5.3 Clinical implications

The clinically relevant interactions summarized in Table 2 emphasize the importance of individualized therapeutic management, particularly in patients receiving polypharmacy or medications with a narrow therapeutic index. Because the strength of evidence supporting individual phytocannabinoid–drug interactions varies considerably, therapeutic decisions should be guided not only by the proposed mechanisms but also by the quality of the available clinical evidence. The evidence-classification framework presented in Figure 2 and applied in the “Evidence level” column of Table 2 facilitates differentiation between interactions supported by controlled clinical studies, human PK investigations, observational studies, case reports, or preclinical evidence, thereby assisting interpretation of the available literature and supporting more informed clinical decision-making. Polypharmacy increases interaction risk not only by increasing the number of potentially affected metabolic pathways, but also by allowing multiple PK and PD mechanisms to occur simultaneously. A cannabinoid may increase exposure to one medication through enzyme inhibition while also potentiating the sedative, hypotensive, cardiovascular, or bleeding-related effects of other concomitant treatments. Risk is therefore determined by the complete medication regimen rather than by an isolated cannabinoid–drug pair. Particular caution is warranted when patients receive medications with narrow therapeutic indices, overlapping CNS effects, or shared dependence on CYP2C19, CYP2C9, CYP3A4, UGT enzymes, or clinically relevant transporters.

**FIGURE 2:**
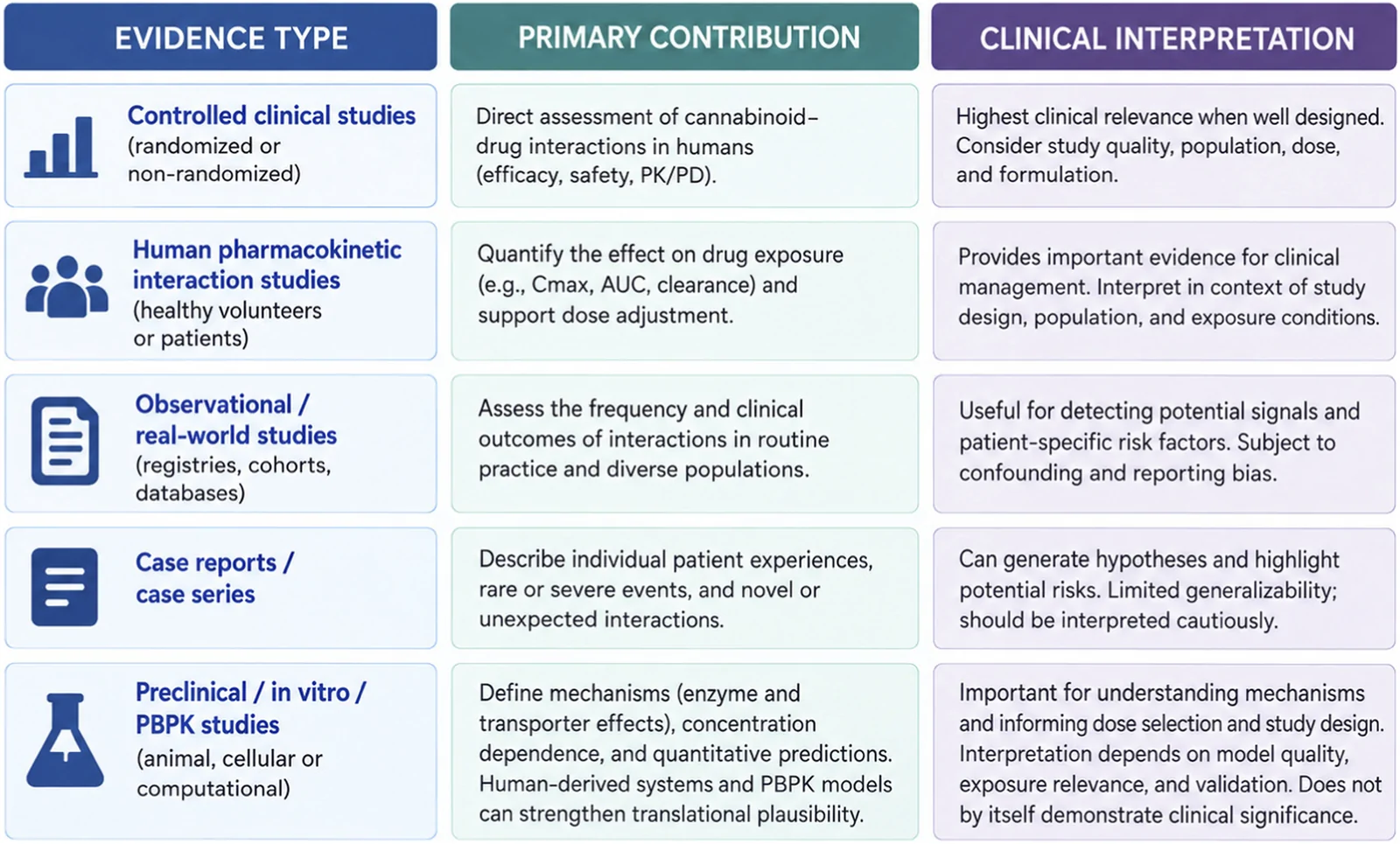
Contribution and interpretation of different types of evidence for phytocannabinoid–drug interactions. The figure presents an interpretative framework for interpreting complementary sources of evidence relevant to phytocannabinoid–drug interactions. Controlled clinical studies, human pharmacokinetic interaction studies, observational or real-world studies, case reports or case series, and preclinical, *in vitro*, or physiologically based pharmacokinetic (PBPK) studies provide complementary forms of evidence regarding interaction effects, mechanisms, and potential clinical relevance. The “Primary Contribution” column summarizes the principal information provided by each evidence type, whereas the “Clinical Interpretation” column highlights its applicability and key interpretative limitations. Preclinical, *in vitro*, and PBPK approaches can provide important mechanistic and quantitative information, particularly when based on human-derived systems or appropriately validated models, but do not by themselves establish clinical significance. Conversely, human evidence should be interpreted in relation to study design, population, exposure conditions, cannabinoid formulation, dose, and route. Accordingly, the evidence types are presented as complementary rather than as a fixed hierarchy. Clinical interpretation should be based on the totality of available evidence, considering directness to human co-administration, consistency and replication, magnitude of effect, relevance of dose, formulation and route, and complementary mechanistic support. Abbreviations: AUC, area under the concentration–time curve; Cmax, maximum plasma concentration; PBPK, physiologically based pharmacokinetic; PD, pharmacodynamic; PK, pharmacokinetic.

Accordingly, clinical assessment should include prescription medicines, over-the-counter products, supplements, *Cannabis* products, route of administration, smoking status, and recent formulation or dose changes. Therapeutic drug monitoring and closer adverse-effect surveillance may be appropriate when clinically validated interactions or narrow-therapeutic-index medications are involved.

Although many cannabinoid-related interactions remain mechanistically predicted or supported primarily by preclinical evidence, several have been documented clinically, particularly those involving CBD and drugs extensively metabolized by cytochrome P450 enzymes (Nachnani et al., 2024; Caicedo et al., 2025). These findings reinforce the need to distinguish predicted interactions from those supported by human evidence when determining the intensity of monitoring and the need for dose adjustment.

Patients receiving polypharmacy, older adults, and individuals with chronic neurological, psychiatric, or cardiovascular disorders represent populations at increased risk for clinically significant interactions and therefore require closer monitoring during cannabinoid therapy (Balachandran et al., 2021; Papakyriakopoulou et al., 2026). Particular caution may also be warranted in patients receiving immune checkpoint inhibitors, as emerging clinical and mechanistic evidence suggests that THC-containing *Cannabis* products may attenuate antitumor immune responses and potentially reduce treatment efficacy. Although current evidence remains limited and heterogeneous, cannabinoid use during immunotherapy should be carefully discussed within the multidisciplinary oncology team and individualized according to the patient’s clinical condition, cannabinoid formulation, and therapeutic goals (Taha et al., 2019; Xiong et al., 2022; Vigano et al., 2025). Similarly, blood pressure monitoring should be considered when cannabinoid-based therapies are initiated or titrated in patients receiving antihypertensive medications, as modest cannabinoid-induced reductions in blood pressure may become clinically significant when combined with concomitant antihypertensive therapy, particularly in older adults and patients receiving multiple antihypertensive agents (Jadoon et al., 2017; Abuhasira et al., 2021; Watanabe et al., 2021; Dragun et al., 2023).

Therapeutic monitoring may be particularly important when cannabinoids are co-administered with medications exhibiting a narrow therapeutic index, including oral anticoagulants, antiseizure medications, immunosuppressants, and certain antiarrhythmics. In these situations, dose adjustment, laboratory monitoring, and close clinical follow-up may be necessary to minimize adverse drug reactions while preserving therapeutic efficacy (Alsherbiny and Li, 2018; Caicedo et al., 2025).

Healthcare professionals should also recognize that the interaction profile may differ substantially according to the formulation employed. Isolated cannabinoids, broad-spectrum extracts, and full-spectrum preparations may exhibit distinct PK and PD characteristics because of differences in phytochemical composition, potentially influencing both therapeutic responses and the magnitude of drug interactions. Nevertheless, the clinical significance of these formulation-dependent differences remains incompletely understood and warrants further investigation (Russo, 2011; Treyer et al., 2023).

Overall, the available evidence supports an individualized approach to cannabinoid-based therapy based on comprehensive medication review, careful dose titration, patient education, and monitoring for both altered drug exposure and additive pharmacological effects (Alsherbiny and Li, 2018; Caicedo et al., 2025). Particular attention should be given to medications with narrow therapeutic indices, CNS depressants, strong metabolic inhibitors or inducers, and changes in *Cannabis* formulation or route of administration. These measures address interaction-related risks, whereas the intrinsic toxicological effects of phytocannabinoids and the susceptibility of specific patient populations require separate consideration, as discussed in the following section.

## 6 Toxicological and population-specific considerations

Beyond PK and PD drug–drug interactions, cannabinoid-based therapies may produce adverse effects arising from the intrinsic pharmacological properties of THC, CBD, other phytocannabinoids, and additional *Cannabis* constituents. The likelihood and severity of these effects vary according to cannabinoid composition, dose, route of administration, treatment duration, concomitant exposures, and patient susceptibility (Kitdumrongthum and Trachootham, 2023; Tudorancea et al., 2026). This section therefore examines acute and chronic toxicological effects, perioperative considerations, contraindications, and populations requiring enhanced clinical caution, while distinguishing intrinsic cannabinoid toxicity from adverse outcomes caused or amplified by drug–drug interactions.

### 6.1 Toxicological considerations and adverse effects

Despite the generally favorable safety profile of cannabinoid-based therapies, phytocannabinoids are not devoid of adverse effects or toxicological concerns. In most clinical settings, adverse effects are dose-dependent and reversible; however, clinically significant complications may occur in susceptible individuals, particularly with high cannabinoid exposure, inappropriate use, or concomitant medications (Gottschling et al., 2020; Balachandran et al., 2021).

Among the major phytocannabinoids, THC is responsible for most dose-limiting adverse effects because of its psychoactive properties mediated primarily through CB1 receptor activation. Frequently reported adverse events include dizziness, somnolence, impaired attention, cognitive slowing, anxiety, tachycardia, orthostatic hypotension, dry mouth, and impaired psychomotor performance. At higher doses, THC may precipitate acute anxiety, panic attacks, paranoia, transient psychotic symptoms, or severe cognitive impairment, particularly in *Cannabis*-naïve individuals or those with underlying psychiatric vulnerability (Huestis, 2007; Crippa et al., 2018; Velayudhan et al., 2021b).

CBD, in contrast, exhibits a considerably more favorable safety profile and lacks the psychotomimetic effects associated with THC. Nevertheless, clinically relevant adverse events have been reported, particularly at higher therapeutic doses such as those used for treatment-resistant epilepsy. The most frequently observed effects include somnolence, fatigue, diarrhea, reduced appetite, and weight loss. Elevations in hepatic transaminases have also been documented, especially when CBD is co-administered with valproate, emphasizing the importance of periodic liver function monitoring during treatment (Millar et al., 2019; Silvestro et al., 2019; Na and Lee, 2025).

Although serious intoxication caused by phytocannabinoids remains relatively uncommon, acute toxicity may occur following accidental ingestion of high-dose *Cannabis* products, concentrated extracts, or edible formulations with delayed onset of action. Clinical manifestations include marked sedation, confusion, ataxia, severe anxiety, hallucinations, cardiovascular alterations, and, in rare cases, seizures or coma. Children are particularly susceptible to accidental intoxication because of their lower body weight and increased CNS sensitivity, frequently requiring emergency medical evaluation following exposure to edible cannabis products (Grant et al., 2018; Gottschling et al., 2020).

Chronic, frequent exposure to THC-containing *Cannabis* products may also result in cannabinoid hyperemesis syndrome, characterized by recurrent episodes of severe nausea, vomiting, and abdominal pain that typically resolve after sustained cessation of THC-containing Cannabis use (Simonetto et al., 2012; Sorensen et al., 2017). Because the syndrome may be mistaken for other gastrointestinal disorders and repeated *Cannabis* use may initially be perceived by patients as antiemetic, recognition of the temporal relationship between chronic exposure and recurrent symptoms is clinically important. The risk appears to be associated predominantly with prolonged and intensive use of THC-containing products rather than with short-term administration of purified CBD.

Perioperative and procedural sedation settings represent another clinically relevant area of concern. Observational studies have associated regular *Cannabis* use with increased requirements for sedative and anesthetic agents, particularly propofol. In a case–control study of 318 patients undergoing endoscopy, *Cannabis* exposure was associated with a higher propofol dose than non-exposure, with daily users requiring greater doses than weekly or monthly users (Imasogie et al., 2021). In a separate retrospective cohort of patients undergoing ambulatory oral and maxillofacial surgery, *Cannabis* users received significantly higher doses of propofol, midazolam, ketamine, and fentanyl than non-users (Ripperger et al., 2023). These findings, which have also been supported by a recent systematic review and meta-analysis (Baker et al., 2025), may reflect PD tolerance, altered CNS responsiveness, or unmeasured confounding and do not establish a defined molecular PK interaction. Nevertheless, *Cannabis* use should be documented during preprocedural assessment and considered when sedative or anesthetic doses are titrated, while prospective studies are needed to clarify the underlying mechanisms and clinical implications.

Toxicological assessment should also consider product quality and composition. Unregulated or inadequately standardized *Cannabis*-derived products may contain cannabinoid concentrations that differ from label claims and may introduce additional risks related to residual solvents, pesticides, heavy metals, microbial contamination, or undeclared pharmacologically active constituents. These concerns are distinct from the intrinsic toxicity of phytocannabinoids but may substantially influence the safety and reproducibility of both therapeutic and nonprescription products. Consequently, findings obtained with regulated, pharmaceutical-grade preparations should not be automatically extrapolated to poorly characterized commercial products.

Long-term safety remains an area requiring further investigation. While chronic therapeutic use of CBD appears to be generally well tolerated, prolonged exposure to THC-rich products has been associated with cognitive impairment, *Cannabis* use disorder, psychiatric complications in susceptible individuals, and possible cardiovascular and respiratory risks depending on the route of administration. Current evidence suggests that many of these long-term adverse outcomes are influenced by cumulative dose, age at exposure, frequency of use, and THC concentration, reinforcing the importance of individualized treatment and careful patient selection (Volkow et al., 2014; National Academies of Sciences, Engineering, and Medicine, 2017; Gottschling et al., 2020).

Finally, toxicological risk should not be evaluated independently of drug–drug interactions. In some clinical situations, adverse outcomes may reflect the combined contribution of intrinsic cannabinoid effects, increased exposure to co-administered medications, and additive pharmacological effects. This distinction is important when assessing causality and determining whether management should involve cannabinoid dose reduction, modification of concomitant therapy, enhanced monitoring, or treatment discontinuation.

### 6.2 Contraindications and vulnerable populations

Although cannabinoid-based therapies may provide clinical benefit in selected circumstances, their use may be contraindicated or require particular caution in specific populations because of physiological vulnerability, altered PK or PD responses, limited clinical evidence, or increased susceptibility to adverse outcomes. Careful assessment of the individual benefit–risk balance is therefore essential before treatment initiation, particularly in patients with substantial comorbidity, organ impairment, psychiatric or cardiovascular vulnerability, or polypharmacy.

Vulnerable populations should be considered in two distinct clinical contexts. The first comprises patients in whom cannabinoid-based therapies may have established or emerging therapeutic applications, such as pediatric patients, older adults, and individuals with hepatic impairment, psychiatric or cardiovascular vulnerability, or polypharmacy, but who exhibit altered PK, increased susceptibility to adverse drug reactions, or a greater likelihood of clinically relevant drug interactions. The second comprises populations in whom cannabinoid exposure is primarily associated with safety and toxicological concerns rather than recognized therapeutic use, particularly pregnant and lactating women, in whom fetal and neonatal exposure and outcomes remains an important concern.

Pregnant and lactating women represent one of the most important populations in whom cannabinoid use should generally be avoided. Phytocannabinoids readily cross the placental barrier and are excreted into breast milk, exposing the developing fetus and neonate during critical periods of neurodevelopment. Experimental and epidemiological studies have associated prenatal *Cannabis* exposure with impaired fetal growth, adverse neonatal outcomes, and potential long-term neurodevelopmental alterations, although confounding factors—including concomitant tobacco and alcohol use—continue to complicate causal interpretation (Richardson et al., 2016; Grant et al., 2018; Navarrete et al., 2020; Inserra et al., 2026). Consequently, current clinical guidelines generally recommend avoiding cannabinoid use during pregnancy and breastfeeding except in exceptional circumstances in which treatment is considered medically necessary following careful specialist assessment of the potential benefits and risks.

In pediatric patients, therapeutic use of phytocannabinoids requires individualized clinical assessment. High-purity CBD has demonstrated a clinically manageable safety profile for the treatment of specific forms of treatment-resistant epilepsy, including Dravet syndrome, Lennox–Gastaut syndrome, and tuberous sclerosis complex, leading to regulatory approval in several countries. Preliminary evidence has also emerged in other pediatric populations. In a retrospective open-label pilot study of 30 children with ASD receiving a CBD-rich oil for 24 weeks, treatment was generally tolerated, including among children undergoing medically supervised reduction or withdrawal of concomitant psychotropic medications; however, the seizure recurrence in two participants during medication tapering and concomitant CBD-rich oil administration underscore the need for careful clinical monitoring (Madruga de Liz et al., 2025). However, evidence supporting cannabinoid use for other pediatric indications remains limited, and long-term safety data are still insufficient. Therefore, cannabinoid therapy in children should be restricted to carefully selected clinical situations and conducted under specialized medical supervision (Devinsky et al., 2018; Thiele et al., 2021; Vega-García et al., 2021).

Enhanced caution is also warranted in individuals with psychiatric or cardiovascular vulnerability. THC-containing products may exacerbate anxiety, paranoia, perceptual disturbances, or psychotic symptoms, particularly in patients with a personal history of psychosis or marked susceptibility to these effects (Fiorentini et al., 2021). Acute increases in heart rate, changes in blood pressure, and orthostatic symptoms may also be clinically relevant in patients with unstable cardiovascular disease or an elevated baseline risk of syncope and falls (Latif and Garg, 2020). In these populations, cannabinoid composition, particularly THC exposure, should be considered carefully, and treatment decisions should be individualized rather than extrapolated from the safety profile of CBD-dominant preparations.

Older adults constitute another population requiring special attention. Age-related physiological changes, including reduced hepatic metabolism, altered body composition, decreased renal function, and increased sensitivity to centrally acting drugs, may substantially modify cannabinoid PK and PD. Furthermore, multimorbidity and polypharmacy markedly increase the likelihood of clinically relevant drug-drug interactions and adverse events, including dizziness, orthostatic hypotension, sedation, cognitive impairment, falls, and cardiovascular complications (Velayudhan et al., 2021b; 2021a; Wolfe et al., 2023). These risks may be amplified by concomitant antihypertensive therapy, particularly in patients receiving multiple blood pressure-lowering agents (Jadoon et al., 2017; Abuhasira et al., 2021; Watanabe et al., 2021; Dragun et al., 2023). Although cannabinoids may provide meaningful therapeutic benefits for selected older patients, treatment should generally follow the principle of “start low and go slow,” with gradual dose titration and close clinical monitoring.

Patients with hepatic impairment require careful evaluation because most phytocannabinoids undergo extensive hepatic metabolism, and impaired liver function may increase systemic exposure and necessitate dose adjustment (Mboumba Bouassa et al., 2022). Evidence in patients with severe renal dysfunction remains limited, warranting cautious use and enhanced monitoring. Particular caution is warranted when cannabinoids are co-administered with drugs that have a narrow therapeutic index, for which even modest changes in exposure may have clinically significant consequences.

Overall, decisions regarding cannabinoid therapy in potentially vulnerable populations should be based on the specific cannabinoid composition, expected clinical benefit, age, physiological status, organ function, psychiatric and cardiovascular risk, comorbidities, and concomitant medications. Where treatment is considered appropriate, careful patient selection, conservative dose titration, and population-specific monitoring are essential to minimize preventable harm.

### 6.3 Future perspectives and research priorities

Despite the remarkable expansion of cannabinoid research over the past 2 decades, important knowledge gaps remain regarding the safe and evidence-based clinical use of phytocannabinoids. While substantial progress has been achieved in understanding cannabinoid pharmacology and therapeutic applications, many aspects related to PK, PD, toxicology, and drug-drug interactions continue to rely on limited clinical evidence. Addressing these gaps will be essential for optimizing cannabinoid-based therapies and supporting their broader integration into clinical practice (National Academies of Sciences, Engineering, and Medicine, 2017).

One of the highest research priorities involves generating high-quality clinical evidence on drug-drug interactions. Although numerous PK interactions have been demonstrated *in vitro* or predicted from metabolic pathways, relatively few have been confirmed in well-designed prospective clinical studies. In this context, real-world evidence studies have emerged as valuable complementary sources of information by capturing the effectiveness and safety of cannabinoid-based therapies under routine clinical practice, particularly in patient populations frequently underrepresented in randomized clinical trials (Papakyriakopoulou et al., 2026). Future investigations should prioritize clinically relevant outcomes, therapeutic drug monitoring, and the evaluation of interaction magnitude across different cannabinoid formulations, doses, and routes of administration.

An additional research priority concerns the interaction between cannabinoids and immune checkpoint inhibitors. Although preliminary clinical observations and mechanistic studies suggest that THC-rich *Cannabis* preparations may impair antitumor immune responses and reduce the efficacy of PD-1 blockade, the available evidence remains limited and sometimes conflicting (Behling-Hess et al., 2025). Prospective clinical studies are needed to determine whether these interactions vary according to cannabinoid composition, dose, route of administration, tumor type, treatment duration, and patient characteristics, while also evaluating their impact on clinically meaningful oncological outcomes and immune-related adverse events.

Another important challenge concerns the increasing availability of *Cannabis*-derived products with highly variable phytochemical compositions. Most current evidence has been generated using purified CBD or THC, whereas considerably less is known about the PK, PD, and toxicological interaction profiles of minor phytocannabinoids such as CBG, CBN, CBC, and THCV, particularly when they are co-administered with conventional medications. Likewise, the potential influence of terpenes and other phytochemicals on drug-metabolizing enzymes, transporters, systemic drug exposure, and PD responses remains insufficiently characterized. Comparative studies of isolated cannabinoids, broad-spectrum formulations, and full-spectrum *Cannabis* extracts are therefore needed to determine whether differences in phytochemical composition modify the magnitude and clinical relevance of drug–drug interactions during cannabinoid–medication co-administration.

Future research should also explore the contribution of pharmacogenomics to phytocannabinoid-related drug–drug interactions, and whether precision-medicine approaches could improve their prediction and clinical management. Genetic polymorphisms affecting cytochrome P450 enzymes, drug transporters, and components of the endocannabinoid system may partially explain the marked interindividual variability observed in therapeutic response and adverse effect profiles. Integrating pharmacogenetic information into cannabinoid prescribing may ultimately contribute to safer and more individualized treatment strategies (Hryhorowicz et al., 2017).

Interactions between phytocannabinoids and psychedelics, including in the context of emerging psychedelic-assisted therapies remain poorly characterized. Naturalistic evidence indicates that concomitant *Cannabis* use is relatively common and suggests that *Cannabis* may be associated with increased intensity and altered qualitative features of psychedelic experiences, including mystical experiences, ego dissolution, visual alterations, and, at higher *Cannabis* doses, more challenging experiences (Kuc et al., 2022). However, these observational findings cannot establish causality or determine whether the observed effects result from PK/PD interactions, expectancy, or other contextual factors. Potential PK interactions are also biologically plausible. CBD inhibits CYP2D6 *in vitro*, whereas CYP2D6 contributes to LSD metabolism and variability in LSD exposure; however, this potential interaction has not been directly evaluated in controlled cannabinoid–psychedelic co-administration studies. At the PD level, CBD has been characterized as a negative allosteric modulator of 5-HT2A receptor signalling *in vitro*, antagonizing LSD-mediated Gq activation while leaving β-arrestin2 recruitment unaffected (Billard et al., 2025). This mechanism could therefore influence the subjective and potentially therapeutic effects of serotonergic psychedelics, although its relevance in humans remains unknown. Evidence of biological crosstalk has also emerged: repeated LSD administration alters brain eCBome mediators in the PFC of mice (Inserra et al., 2023) while ayahuasca administration modifies circulating endocannabinoid and eCBome-related lipid concentrations in humans (dos Santos et al., 2022; Madrid-Gambin et al., 2026), supporting the biological plausibility of bidirectional crosstalk between serotonergic psychedelic signaling and the ECS/eCBome. These findings do not, however, demonstrate a direct drug–drug interaction. Controlled clinical studies directly evaluating cannabinoid–psychedelic PK and PD interactions are lacking. Potential interactive effects on anxiety, perception, cognition, cardiovascular responses, and psychiatric vulnerability warrant systematic investigation.

Population-specific interaction research should distinguish among groups in whom specific cannabinoid medicines are used therapeutically, groups in whom cannabinoid exposure may be harmful, and groups for whom interaction evidence remains insufficient. In paediatric patients receiving purified CBD for approved epilepsy indications and in older adults treated for selected conditions, prospective studies should quantify interactions with commonly co-administered medications and define appropriate monitoring strategies. In pregnancy and lactation, research should primarily clarify maternal, fetal, and neonatal risks associated with cannabinoid exposure and concomitant medication use rather than assume therapeutic applicability. Older adults, individuals with hepatic impairment, patients with multiple comorbidities, and other populations frequently exposed to polypharmacy also remain underrepresented in prospective drug–drug interaction studies. Future studies should therefore evaluate specific cannabinoid compounds and formulations rather than treating cannabinoid exposure as a uniform intervention.

Recent advances in analytical chemistry, therapeutic drug monitoring, real-world evidence, and artificial intelligence-based predictive models are expected to improve the identification, prediction, and management of cannabinoid-related drug interactions. Likewise, harmonization of international regulatory frameworks and improved standardization of *Cannabis*-derived medicinal products will be fundamental to ensuring reproducibility across clinical studies investigating cannabinoid-based therapies.

Ultimately, advancing the understanding and clinical management of phytocannabinoid-related drug–drug interactions requires an integrated approach that combines basic pharmacology, clinical pharmacology, toxicology, pharmacogenomics, and clinical research. Such multidisciplinary efforts will be essential to distinguish predicted interactions from those of established clinical relevance, identify patient- and treatment-specific risk factors, refine dose-adjustment and therapeutic drug-monitoring strategies, and support the safe, effective, and individualized co-administration of phytocannabinoid-based therapies with conventional medications across diverse clinical settings.

## 7 Conclusion

The growing medical use of cannabinoid-based therapies has increased their relevance in the management of selected neurological, psychiatric, inflammatory, and chronic pain conditions. At the same time, their expanding use has raised important concerns regarding drug interactions, toxicological risks, and patient safety.

Current evidence demonstrates that clinically established phytocannabinoid-drug interactions arise predominantly from modulation of drug-metabolizing enzymes, particularly members of the cytochrome P450 family, as well as from additive PD effects on the CNS and other physiological systems. These clinically established interactions have been most consistently documented for antiseizure medications, immunosuppressants, CNS depressants, and alcohol, whereas anticoagulant interactions, although clinically important, remain supported largely by case-based and mechanistic evidence rather than controlled interaction studies. In addition, mechanistic, *in vitro*, and emerging clinical evidence suggests the potential for numerous predicted interactions involving other therapeutic classes; however, many of these remain insufficiently investigated in controlled clinical studies. Although CBD has emerged as the phytocannabinoid most frequently associated with clinically significant PK interactions, the increasing therapeutic use of other cannabinoids and *Cannabis*-derived formulations reinforces the need for broader investigation of their individual and combined interaction profiles. Evidence regarding CBD- and THC-related interactions has expanded considerably; however, the interaction profiles of minor phytocannabinoids, terpenes, and full-spectrum formulations remain incompletely characterized, and many proposed interactions await confirmation in clinical studies. Across therapeutic classes, mechanistic plausibility should therefore be distinguished from demonstrated clinical significance when interpreting interaction risk.

Despite substantial advances in cannabinoid research, important uncertainties remain regarding the long-term safety of phytocannabinoids, the clinical significance of many predicted drug interactions, and the influence of pharmacogenetic variability, cannabinoid formulation, route of administration, and patient-specific characteristics on therapeutic outcomes. These limitations are particularly relevant in vulnerable populations. In some groups, including older adults, pediatric patients, individuals with hepatic impairment, and patients receiving multiple medications, cannabinoid-based therapies may require individualized dosing and careful monitoring because of increased susceptibility to clinically relevant drug interactions. In contrast, during pregnancy and lactation, the principal concern relates to fetal and neonatal exposure and the limited evidence supporting safety, rather than to therapeutic drug interactions themselves.

Future progress in cannabinoid therapeutics will depend on the integration of mechanistic and clinical pharmacology, toxicology, pharmacogenomics, therapeutic drug monitoring, and clinical and preclinical interaction studies to better identify, quantify, and manage clinically relevant phytocannabinoid–drug interactions. Accordingly, comprehensive risk assessment should integrate PK, PD, and toxicological evidence to better predict clinically relevant phytocannabinoid–drug interactions and optimize individualized cannabinoid-based therapies. Such an integrated approach will not only improve the prediction and management of cannabinoid-related drug interactions but also improve risk stratification, inform monitoring and dose-adjustment strategies, and support safer use of cannabinoid-based therapies in patients receiving concomitant medications.

Ultimately, the transition from empirical cannabinoid prescribing toward precision cannabinoid medicine represents one of the greatest challenges-and opportunities-for the coming years. Achieving this goal will require a deeper understanding of the complex interactions between phytocannabinoids, patient-specific biological characteristics, and concomitant pharmacotherapy, thereby enabling evidence-based, personalized, and safer cannabinoid treatment across diverse clinical settings.
